# Redescription of Female *Laelaps nuttalli* Hirst, 1915 (Acari: Dermanyssoidea: Laelapidae) with Emphasis on Its Gnathosoma, Sense Organs and Pulvilli

**DOI:** 10.5402/2013/642350

**Published:** 2013-08-27

**Authors:** Ashraf Ahmed M. E. Montasser

**Affiliations:** ^1^Biology Department, College of Science and Humanity Studies, Salman bin Abdulaziz University, P.O. Box 11942, AlKharj, Saudi Arabia; ^2^Zoology Department, Faculty of Science, Ain Shams University, P.O. Box 11566, Cairo, Egypt

## Abstract

The present scanning electron microscopic (SEM) study includes the redescription of female *Laelaps nuttalli* with emphasis on its gnathosoma and pulvilli which were rarely described in superfamily Dermanyssoidea. Chaetotaxy of dorsal shield revealed 40 pairs of setae, 22 on prosoma and 18 on opisthosoma. Epigynial plate carried 4 pairs of setae. Gnathosoma consisted of long basis capituli carrying median hypostome and 2 lateral pedipalps. Hypostome had dorsal labrum of 2 lobes covered with minute papillae, 2 lateral 3-segmented chelicerae, and ventral labium carrying 2 median lobes with laciniae and 2 lateral club-like lobes. Function of labrum papillae might be chemosensory while labium lobules might be mechanical, preventing solid material from entering the oral cavity. Palpal and foreleg tarsal organs comprised 10 and 15 sensilla, respectively. Sensilla of palpal organ were mostly chemoreceptors while those of tarsal organ were probably mechanoreceptors. Each pulvillus terminated with 2 medioventral claws and integumental folds beside longitudinal folds.

## 1. Introduction

Acari are immensely important group of ectoparasites affecting human, animals, and plants. The mite, *Laelaps nuttalli,* occurs throughout the world as a parasite of the black rat *Rattus rattus*, the brown rat *R. norvegicus* and occasionally of other rodents and small mammals [1]. *L. nuttalli* received the attention of several authors since the mid-twentieth century. Studies on *L. nuttalli* were mainly concerned with surveys and host-parasite relationships [2–18]. Recently, *L. nuttalli* was also collected from bats in Malaysia [19]. 

Considerable literatures were encountered in the redescription of mite species with SEM [20–26]. SEM was also useful in describing certain parts of mites such as the gnathosoma [27–30], palpal organ [31, 32], and foreleg tarsal organ [32–34]. Description of *L. nuttalli* was only investigated with LM in the studies of Keegan [1] and Tipton [35].

The present SEM study proffers redescription of *L. nuttalli *with particular attention to the undescribed details of idiosoma, gnathosoma, peritreme, sense organs, and pulvilli. These structures were rarely described in superfamily Dermanyssoidea which includes 15 families; most of them are parasites and disease carriers. Of these families, species of Dermanyssidae, Laelapidae, and Macronyssidae are proven transmitters of diseases in birds, reptiles, and mammals including man [36]. Detailed morphology of the gnathosoma, peritreme, and pulvilli is an important step towards elucidation of the feeding behavior, respiration, and attachment mechanism, respectively, of this mite and hence its pathology. Sense organs such as palpal or tarsal organs are of primary importance in relation to orientation responses to humidity, temperature, and chemical stimuli. 

## 2. Materials and Methods

*L. nuttalli* of the present study were recovered from *R. rattus* collected from Bilbeis area, about 60 km northeast of Cairo, Egypt. The study area and method of collection were described by Soliman et al. [13, 14]. Mites were separately processed for mounting and identified according to Keegan [1], Tipton [35], and Krantz [36].

*L. nuttalli* were washed several times using saline solution to remove debris. Specimens were fixed in 2.5% glutaraldehyde mixed in phosphate buffer solution (PBS) at a pH of 7.4 at 4°C for 24 h. They were then rinsed twice with PBS at 10 min intervals. Specimens were then treated with 1% osmium tetraoxide at room temperature for 1 day for post-fixation. This was followed by rinsing twice with PBS and dehydrating with alcohol. To replace water in mites with alcohol, they were subjected to increasing concentrations of ethanol as follows: 30%, 50%, 70%, 80%, 90%, and 95% for 15 min each. They were then placed in absolute alcohol for 10 min for 2 changes. Finally, they were subjected to critical point drying in order to complete the dehydration process [37]. In order to view specimens, they were first attached with double-sided carbon tape to aluminum stubs so that they could be coated with gold in a sputter-coating apparatus (JEOL JFC-1200). The surface topography of specimens was viewed at 25 kV in a JEOL-JSM5600 scanning electron microscope (Japan). 

## 3. Results

**Figure 1:**
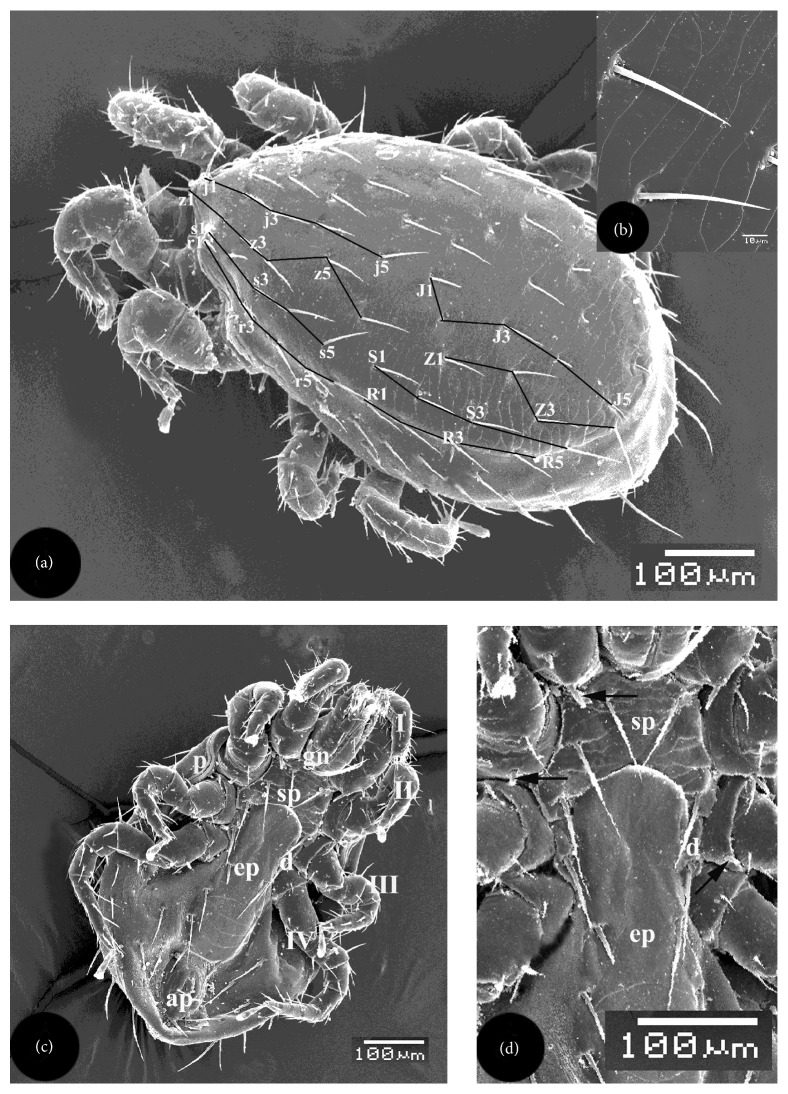
Scanning electron micrographs of dorsal and ventral surfaces of female *Laelaps nuttalli.* (a) Chaetotaxy of the dorsal shield showing distribution of 40 pairs of setae. Black lines connect setal sockets in each series which include j1–j5, z1–z6, s1–s5, r1–r6, J1–J5, Z1–Z4, S1–S4, and R1–R5. (b) Posterior region of dorsal shield showing long pointed setae and considerable striations. (c) Whole ventral side showing gnathosoma (gn), sternal plate (sp), epigynial plate (ep), endapodal plate (d), anal plate (ap), peritreme (p), and 4 pairs of legs (I–IV). (d) Sternal plate (sp) carrying 3 pairs of long pointed setae, epigynial plate (ep) carrying 4 pairs of pointed setae, endapodal plate (d) carrying 1 long pointed seta, and coxae of the first three legs carrying short stout posteriorly directed spurs (arrows).

Female *L. nuttalli *was medium sized, oval, and almost covered with sculptured dorsal shield (Figure 1(a)). Lateral shoulders were markedly noticed on anterior 1/6 of the dorsal surface (Figure 1(a)). Body was 602.7 *μ*m in length and 386.9 *μ*m in width. Dorsal shield was posteriorly rounded and rarely ornamented with transverse overlapping striations (Figure 1(a)). Posterior striations were considerably noticed (Figure 1(b)). Dorsal shield measured 546 *μ*m in length and 302.7 *μ*m in width. It had 40 pairs of setae, 22 on the prosoma and 18 on the opisthosoma (Figure 1(a)). Prosomal setae included 4 series, namely, j1–j5, z1–z6, s1–s5, and r1–r6. Opisthosomal setae also included 4 series, namely, J1–J5, Z1–Z4, S1–S4, and R1–R5. Nomenclature of setae was according to Krantz [36]. The majority of setae were simple and slender with fine longitudinal ribs (Figure 1(b)). Most setae were long (48.7–59.5 *μ*m), while few setae as r5 were small (16.2 *μ*m). 

Ventrally, *L. nuttalli* had considerably sclerotized plates, namely, sternal, epigynial, anal, and endapodal plates (Figures 1(c) and 1(d)). Sternal plate anterior margin was almost straight and just behind the gnathosoma while its posterior margin was concave and slightly covered with the anterior margin of the epigynial plate (Figure 1(d)). The posterolateral corners of sternal plate project distinctly between coxae II and III. Sternal plate carried 3 pairs of long pointed setae; the anterior setae were shorter than the others. Length of sternal plate (77.2 *μ*m at mid-line) was much shorter than its width (117.6 *μ*m at the level of the second setae). No pores were noticed on that plate (Figure 1(d)). Two polygonal endapodal plates were located posterolateral to the sternal plate and adjacent to the anterior margin of the epigynial plate (Figures 1(c) and 1(d)). Each plate carried a long pointed seta. Epigynial plate, or genitoventral shield, extended between sternal and anal plates, measured 276.9 *μ*m long and 107.7 *μ*m wide (Figure 1(c)). It was broader behind coxae IV and its posterior margin was straight that separated from anal plate by a wide area of integument. Epigynial plate had 4 pairs of pointed setae of more or less equal length (Figure 1(c)). Anal plate was longer (96.2 *μ*m) than wide (61.5 *μ*m) (Figure 1(c)). It was almost oval in shape with narrow posterior end. Longitudinal anal opening was guarded with 2 rectangular leaves which in turn were surrounded with elevated oval integument (Figure 1(c)). Anal plate carried 3 setae; setae of the anterior pair were located posterolateral to anal leaves and approximately half as long as the posterior unpaired seta which is located behind the anal plate (Figure 1(c)). All the above plates were faintly ornamented. Integument adjacent to epigynial and anal plates contained horizontal and longitudinal striations (Figure 1(c)). About 7 pairs of setae, measuring 30.8–69.2 *μ*m, were noticed on the soft integument of ventral side outside anal and epigynial plates (Figure 1(c)).

**Figure 2:**
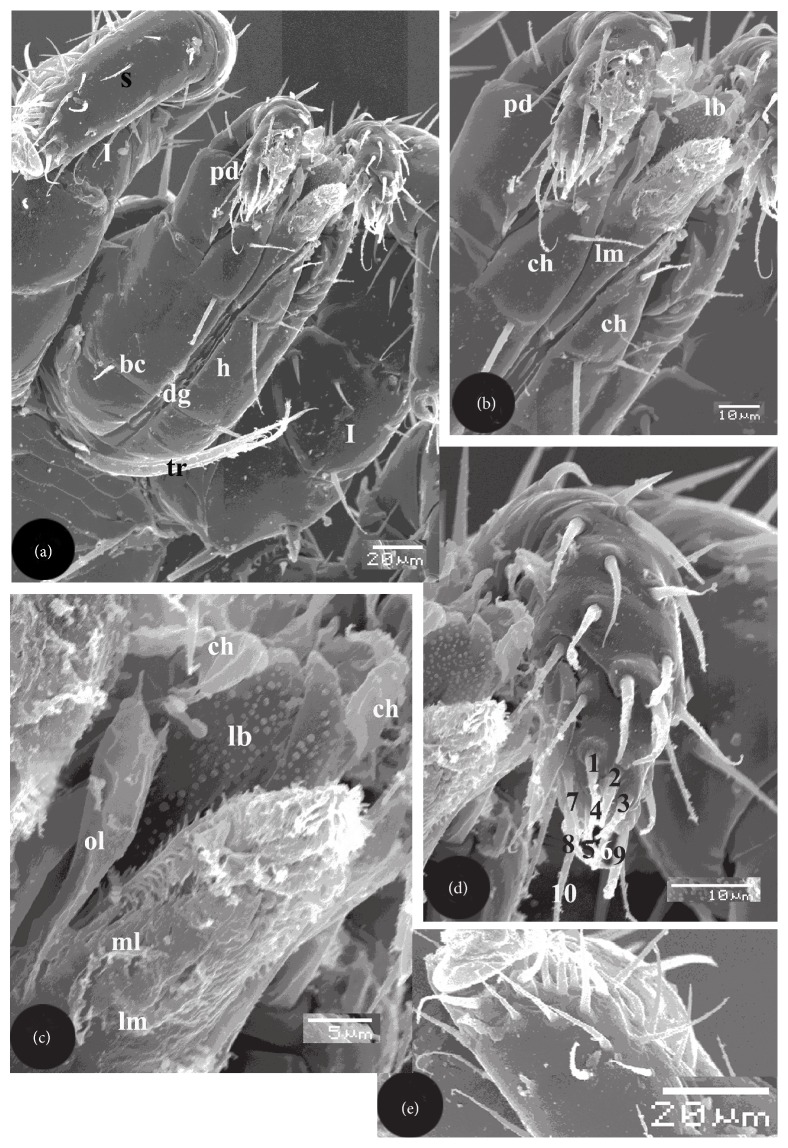
Scanning electron micrographs of gnathosoma and sense organs of female *L. nuttalli*. (a) Whole gnathosoma consisting of long basis capituli (bc) carrying median hypostome (h) and 2 lateral pedipalps (pd). Tritosternum (tr) with 2 laciniae located below the deutoternal groove (dg) on the ventral side of the basis capituli. I = first leg, s = tarsus. Arrows showing spines in the deutosternal groove. (b) Hypostome carrying dorsal labrum (lb), ventral labium (lm), and 2 lateral chelicerae (ch). Pd = pedipalp. (c) As in (b) showing labrum (lb) consisting of 2 lobes, each carrying minute papillae, terminal segments of chelicerae (ch), and labium consisting of club-shaped outer lobes (ol) and 2 median lobes (mL) carrying laciniae (arrows). (d) Palpal organ (po) consisting of 10 terminal setae (1–10). (e) Foreleg tarsal organ (to) consisting of 15 terminal setae.

The gnathosoma, or capitulum, consisted of long basis capituli carrying median hypostome and 2 lateral pedipalps (Figure 2(a)). Long well-developed tritosternum between 2 small triangular integumental folds was originated at the base of the basis capituli (Figure 2(a)). Tritosternal base was longer than the broad one, undivided, and bifurcated into 2 long laciniae (Figure 2(a)); each was anastomosed into numerous long processes (Figure 2(a)). Deutosternl groove was a longitudinal median groove noticed on the ventral side of the basis capituli above the tritosternum (Figure 2(a)). The groove contained 6 horizontal rows; each had 2 spines (Figure 2(a)). Hypostome carried dorsal labrum, 2 lateral chelicerae, and ventral labium (Figure 2(a)). 

Labrum is well developed and consisted of 2 lobes; each had numerous minute less electron dense papillae (Figure 2(c)). Chelicera consisted of 3 segments; the first represented the base which carried other 2 segments (Figures 2(a)–2(c)). The first segment carried 2 ventral setae (Figure 2(b)). The terminal segments form a chela of 2 elongate edentate digits (Figure 2(c)). Labium appeared as inverted isosceles triangle, its tip observed in front of the deutosternal groove of basis capituli (Figures 2(a) and 2(b)). Base of labium carried 2 median and 2 lateral lobes (Figure 2(c)). Each median lobe carried externally allocated elongated lacinia (Figure 2(c)). Lateral lobes appeared as club-like lobes where they were narrow at their bases and broad terminally (Figure 2(c)).

Sense organs in *L. nuttalli* included the palpal and foreleg tarsal organs (Figures 2(a)–2(e)). SEM showed a sensorial cluster on the tip of the terminal segment of the 6-segmented pedipalp (Figures 2(a), 2(b), and 2(d)). This cluster, or palpal organ, comprised 10 setiform sensilla which can be characterized according to their shape, size, tip and socket (Figure 2(d)). Sensilla 1–3 were the smallest, peg-like with sharp tips and with sockets. Sensilla 4–6 were thin, straight, and longer than sensilla 1–3 and without sockets. Sensilla 7–10 were the largest and had apparent sockets. The base of the socket was raised (Figure 2(d)). The tarsal organ was dorsally located on the tip of the foreleg tarsus (Figures 2(a) and 2(e)). It comprised about 15 setiform sensilla that were greatly similar to each other either in shape or size (Figure 2(e)).

**Figure 3:**
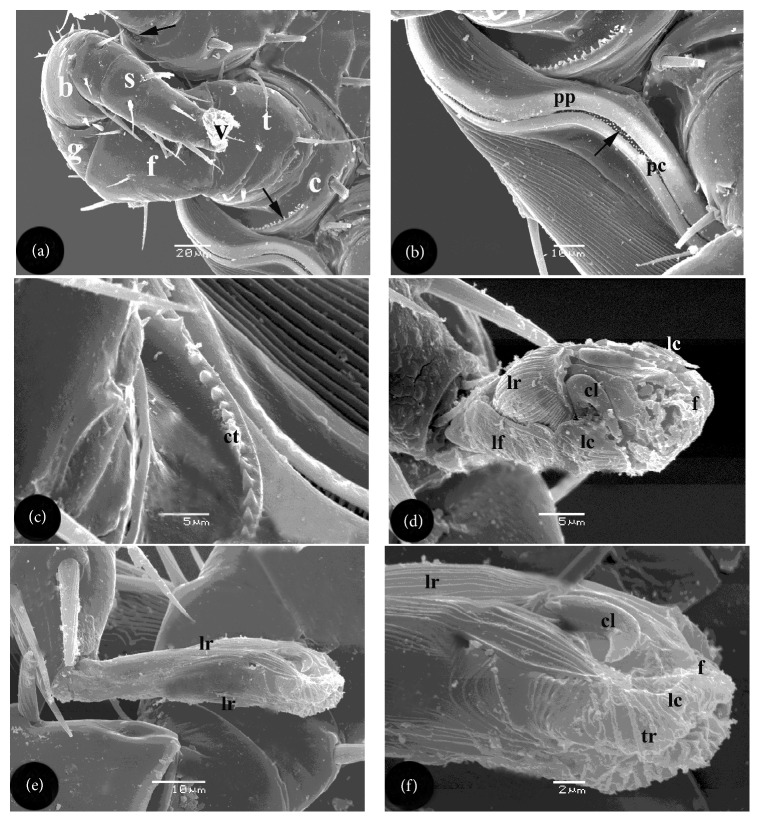
Scanning electron micrographs of peritreme and pulvilli of female *L. nuttalli*. (a) The second leg, consisting of 6 segments, namely, coxa (c), trochanter (t), femur (f), genu (g), tibia (b), and tarsus (s) terminating with pulvillus (v), beside the peritreme. The coxa carries a row of minute conical tubercles (arrow) near the peritremal plate (pp). (b) Peritremal plate (pp) surrounding peritremal canal (pc) which contains minute papillae. (c) Higher magnification of the row of minute conical tubercles (ct) on the coxa. (d) Pulvillus of the first leg carrying 2 curved claws (cl), ventral longitudinal ribs (lr), two lateral laciniae (lc), and overlapping terminal integumental folds (f). (e) Pulvillus of 2nd–4th legs showing longitudinal integumental ridges (lr) on dorsal and ventral sides. (f) As in (e) showing terminal medioventral claws (cl), lateral laciniae (lc), and transverse ridges (tr).

 Peritreme extended anteriorly to the margin of coxa II from an oval pit situated laterally between the third and the fourth coxae (Figure 1(c)). Peritreme was a hardly sclerotized plate surrounding the pit and the anterior peritremal canal (Figures 1(b), 3(a), and 3(b)). The pit was greatly concealed by segments of leg III (Figure 1(b)). Peritremal canal contained 1 or 2 rows of minute papillae (Figure 3(b)). 

Four pairs of walking legs were located on the ventral surface; each carried coarse medium-long sized setae and divided into 6 segments (Figures 1(c) and 3(a)). Beginning with the most proximal, they were coxa, trochanter, femur, genu, tibia, and tarsus (Figure 3(a)). The latter hanged terminal pulvillus. The respective lengths of legs (including pulvilli) were as follows: I-288 *μ*m, II-288 *μ*m, III-344 *μ*m, and IV-504 *μ*m. Coxae of the first 3 legs were adjacent to the sternal plate while those of the 4th one were adjacent to the anterior 1/3 of the epigynial plate. Segments of legs I and II were somewhat uniform in size. Posterior margin of each of I–III coxae carried a short, stout, posteriorly directed, ventral spur (Figure 1(d)). Each of the four pairs of coxae carried a row of minute tapered conical tubercles on their posteroventral edges (Figures 3(a)–3(c)). Pulvillus of the first leg had rough surface of longitudinal ribs and two curved claws on the ventral side and overlapping integumental folds on terminal and dorsal sides (Figure 3(d)). Two lateral folds were observed at the base of that pulvillus (Figure 3(d)). Each pulvillus of second, third, and fourth legs carried a pair of ventroterminal curved claws (Figures 3(e) and 3(f)). Each pulvillus carried a pair of lateral comb-like laciniae (Figures 3(d) and 3(f)). Integument of the pulvilli 2–4 had longitudinal ridges on ventral and dorsal sides in addition to terminal transverse ridges and integumental folds (Figures 3(e) and 3(f)). 

## 4. Discussion

*L. nuttalli* in previous survey studies, which represent the major work carried out on this mite, recorded the highest infestation rates and densities among ectoparasites infesting the domestic rodents. *R. rattus* and *R. norvegicus*. Accordingly, it might be concluded that *L. nuttalli* almost share the transmission and/or epidemiology of some pathogens of these rodents. Generally, the vector capacity of *L. nuttalli *is still unknown and it requires further studies to identify such pathogens. 

Redescription of *L. nuttalli* with SEM in the present study greatly supported the diagnostic characters previously mentioned by Keegan [1] and Tipton [35]. These characters included the body length, shape of sternal, epigynial, and anal plates and spurs on coxae I–III. Chaetotaxy of the dorsal shield of *L. nuttalli* has been described for the first time in the present study. It revealed 40 pairs of setae that considerably resembled the chaetotactic pattern of the genera *Haemolaelaps* and *Laelaps* described by Costa [38]. Dorsal shield in acarines provides a degree of protection from desiccation and predation [36]. 

Gnathosomalstructure of *L. nuttalli* consisting of median hypostome and 2 lateral pedipalps on long basis capituli, ventral tritosternum, and the 3-segmented chelicerae was greatly matched with those of the generalized gamasid mite described by Krantz [36]. The terminal 2 segments of each chelicera were common in blood sucking mesostigmatic [36] and asitigmatic mites [30] and were specialized for piercing host tissues. Wernz and Krantz [39] suggested the role of the tritosternum as a fluid transporter, directing prey fluids to the prebuccal region. Tipton [35] mentioned that labrum of *L. nuttalli* was only lanceolate and grooved to the apex. Krantz [36] mentioned that labrum dorsally bordered the buccal cavity and functions as a prepharyngeal valve preventing loss of food. Minute papillae covering the labrum and the club-shaped lateral lobes and median lobes carrying laciniae forming the labium in the present study have not been previously described and their function was uncertain. Function of labrum papillae might be chemosensory while lateral and median lobes of labium might be mechanical, preventing any solid material from entering the oral cavity. 

Palpi are simple sensory appendages equipped, with terminal chemosensory sensilla, that aid the acarine in locating its food [36]. In the present study, each pedipalp consisted of 6 segments terminated with palpal organ which moved freely via articulations between palpal segments. This holds up its primary function for food acquisition. This greatly resembled that observed in gamasid mites and argasid ticks while in ixodids, palpal organ was located in a depression of the 3rd palpal segment [36]. In *L. nuttalli*, palpal organ consisted of 10 setiform sensilla forming a cluster terminal to the pedipalp. Six of these sensilla were small-medium sized, and broad with pointed tips. These features support the olfactory or chemosensory function as described in gamasid mites [31, 32]. The longest 4 sensilla probably had a mechanoreceptor function as mentioned in the above studies. Tarsus I carried a cluster of 15 sensilla, and almost all of them were small with pointed tips. Function of these sensilla is most likely to be chemoreceptive. This was in great accordance with those described in other gamasid mites [32, 33].

SEM of the peritreme revealed an oval pit and anterior canal reaching coxa II. Peritremal canal contained minute papillae. This was in great agreement with that described in *Ornithonyssus bacoti* [23] and *Echinolaelaps echidninus* [25]. Papillae appeared to have delicate walls which may share in the process of respiration in the present mite as they might adjoin both the body cavity and the atmospheric air. Further investigation including transmission electron microscopic study is required to support this view.

It is generally accepted that the claws are used to interlock with macroscopical asperities of the substrate. If the diameter of the substrate asperities is lower or equal to the diameter of the claw tip and the substrate is stiff enough to prevent its penetration by claws, the claws slide over the substrate [40]. In the present study, claws were nearly small, curved, and ventrally located in front of longitudinal integumental ridges. Accordingly, the probable function of claws in *L. nuttalli* is supporting the attachment with the host skin. The comb-like plates noticed lateral to pulvilli may contribute to removing debris at the attachment site of the host skin, particularly during the detachment of the mite. Beside these plates, integumental folds and the claws were observed terminal to each pulvillus. These folds may cause the mite to lean on the host skin particularly during the feeding process.
